# Supplementary figures and images for: Willingness, motivators and barriers to time bank participation: A scoping review
Source: PLoS One. 2026 Apr 1;21(4):e0322760. doi: 10.1371/journal.pone.0322760 (PMC13042674; doi:10.1371/journal.pone.0322760)

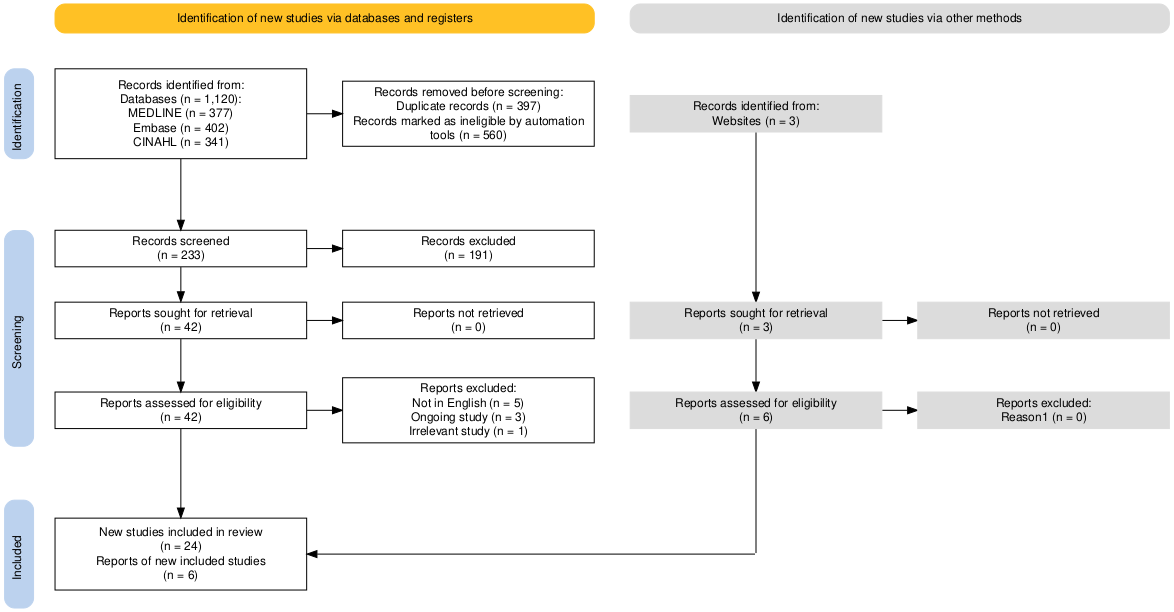

Supplement: S1 Fig — https://estech.shinyapps.io/prisma_fowdiagram/. (DOCX) [file pone.0322760.s001.docx]
