# Supplementary material for: Willingness, motivators and barriers to time bank participation: A scoping review
Source: PLoS One. 2026 Apr 1;21(4):e0322760. doi: 10.1371/journal.pone.0322760 (PMC13042674; doi:10.1371/journal.pone.0322760)
Supplement: S1 Table — (DOCX) [file pone.0322760.s003.docx]

| **No.** | **Author/Year** | **Aim/Objective** | | **Location** | **Sampling Method/Sample** | **Data Collection** | **Design/ Measurement** | | **Findings** |
| --- | --- | --- | --- | --- | --- | --- | --- | --- | --- |
| 1. | Hao et al. (2024) | To examine the impact of physical and mental quality of life on Chinese older adults’ willingness to participate in healthcare time banking. | | China | Stratified  N = 2147 | Self-administered questionnaire | Cross-sectional  Researcher developed questionnaire | | 58% of older adult were willing to participate in a healthcare time banking program. There was a significant positive correlation between physical QoL and willingness of older adults to participate time banking (r=0.532, p<0.01). Mental QoL had significant negative correlation with willingness of older adults to participate time banking (r=-0.61, p<0.01). Females, urban residents and youngest older adults were more likely to participate in time banking. |
| 2. | Wu et al. (2024) | To identify factors influencing university students’ participation in time banking for older adults. | | China | Convenience  N = 254 | Online questionnaire | Cross-sectional  Researcher developed questionnaire | | A significant majority (82.67%) of participants expressed willingness to engage in volunteer services for older adults. Factor analysis uncovered six influential factors explaining 62.55% of the variance. Logistic regression highlighted four key determinants of students’ willingness: value judgment (OR = 4.392, CI = 2.897–6.658), social support (OR = 1.262, CI = 0.938–1.975), social influence (OR = 1.777, CI = 1.598–3.799), and socioeconomic conditions (OR = 1.174, CI = 1.891–3.046). |
| 3. | Cui et. al (2023) | To analyze nurses’ intention and influencing factors to participate in time bank for older adults with disabilities | | China | Convenience  N = 1998 | Self-administered and online questionnaire | Cross-sectional  Researched developed questionnaire | | 59.6% of nurses were willing to participate in volunteers care for older adults with disabilities and the willingness of nurses to participate in volunteer care was above medium level. The more positive attitudes, the more support, the fewer the obstacles the greater the intention of the nurses to participate. |
|  | | | | | | | | | |
|  | | | | | | | | | |
|  | | | | | | | | | |
| Table 1, continued. | | | | | | | | | |
| **No.** | **Author/Year** | **Aim** | | **Location** | **Sampling Method/Sample** | **Data Collection** | | **Design/**  **Measurement** | **Findings** |
| 4. | Aksin et al. (2023) | To assess the intention to engage in time bank among caregivers of older people with dementia. | | Turkey | Convenience  N = 184 | Self-administered questionnaire | | Cross-sectional  Zarit caregiver burden, WHO brief QoL scale and chronic stress scale | Caregivers experiencing higher caregiver burden showed higher intention to engage peer-to-peer exchange services (0.079, p<0.001). Caregivers who can share expertise (0.579, p=0.021), caregiver technology affinity (0.458, p=0.04) and ability and openness to seek professional help for psychological diagnoses (1.595, p=0.012) all demonstrate a higher intention to engage in peer-to-peer exchange. |
| 5. | Yao et al. (2022) | To assess the influence factors of the mutual‐support willingness | | China | Convenience  N = 2167 | Self-administered questionnaire | | Cross-sectional  Researcher developed questionnaire | Factors influencing the mutual‐support willingness and needs included individual characteristics, family environment, and so on. And the rural elderly’s demand for mutual‐support is at a relatively high level. |
| 6. | Kakar et al. (2020) | To investigate the impacts of utilitarian value, hedonic value and social value on the intention of members to participate in time-based banking. | | United States | Convenience  N = 815 | Online questionnaire | | Cross-sectional  Researcher developed questionnaire | The results of the study from across web-based time banks show that time banks provide all three types of value to its participants. However, while the social value of time banks had the highest impact on providers of service, utilitarian value had the highest impact on receivers of services using time banks. |
| 7. | Shao et al. (2020) | To determine the community’s willingness and perception of time bank in Hangzhou. | | China | Convenience  N = 892 | Online questionnaire | | Cross-sectional  Researcher developed questionnaire | 62.7% of respondents were “very willing” and “willing” to participate. There was significant different in respondents’ gender, age, education level with willingness to participate (p<0.05). In terms of “whether or not for community elderly volunteer to provide services” 61.9% were in great need and 23.1% were in general and 17.3% were in little to very unnecessary. |
| Table 1, continued. | | | | | | | | | |
| **No.** | **Author/Year** | **Aim** | | **Location** | **Sampling Method/Sample** | **Data Collection** | | **Design/**  **Measurement** | **Findings** |
| 8. | Yuan et al. (2019) | To examine the users perceive timebank offers and requests differently and how they influence actual use. | | United States | Convenience  N = 220 | Online questionnaire | | Cross-sectional  Researcher developed questionnaire | Perceived ease of use of time banking platforms was positively associated with positive attitudes toward both requests and offers, whereas perceived usefulness was negatively associated with them. Having positive attitudes toward requests was important to elicit its behavioural intention but not offers. |
| 9. | Sasananan et al. (2019) | To explores the interest in joining time banking in Bangkok | | Thailand | Convenience  N = 400 | Self-administered and online questionnaire | | Cross-sectional  Researcher developed questionnaire | Greatest willingness to participate in time banking were found among the younger generation at the age between 18-23 years old (71%) and the lowest willingness were among the older people (34%) |
| 10. | Akter & Abonty  (2019) | To examine the impact of drivers/barriers and personal values and how these are connected to the commitment | | United States  New Zealand  India | Convenience  N = 155 | Online questionnaire | | Cross-sectional  Researcher developed questionnaire | Values play significant role to shape commitment to timebank and commitment and personal values has relationship with drivers and barriers of participation in timebank. |
| 11. | Ezulike et al. (2024) | To explore older adults’ motives for informal caregiving | | Nigeria | Purposive  N = 30 | Semi-structured interview | | Qualitative phenomenological  Thematic analysis | Themes:   1. reciprocity of kindness 2. altruism 3. a sense of moral responsibility 4. eagerness for peaceful longevity |
| 14. | Lu et al. (2023) | To describe the features and explore the underlying mechanism of engaging older adult as volunteers. | | China | Snowball  N = 18 | Semi-structured focus group interview | | Qualitative Case study  Thematic analysis | Three themes emerged as factors that facilitate volunteer engagement:   1. Strong cross-sector collaboration 2. Meaningful in voluntary work 3. Coproduction environment |
| Table 1, continued. | | | | |  |  | |  |  |
| **No.** | **Author/Year** | **Aim** | | **Location** | **Sampling Method/Sample** | **Data Collection** | | **Design/**  **Measurement** | **Findings** |
| 12. | Verma et al. (2023) | To explores the scope of services and challenges in implementing Time Bank in India | | India | Convenience  N = 20 | Semi-structured telephone interview | | Qualitative  Thematic analysis | Themes:   1. Services 2. Challenges 3. Limitations of time bank 4. Factors affecting help-seeking behaviour   Participants expressed interest in offering a wide range of services, from household tasks to professional services. However, challenges and limitations were also identified, such as a lack of awareness and understanding of the concept, social stigma, and concerns about trust, privacy, and safety issues. |
| 13. | Jiao et al. (2023) | To investigate the motivation of members to participate in web-based time banks from a service requirement perspective. | | China | Convenience  N = 12399 | Data-driven  Open coding, spindle coding and selective coding | | Grounded theory and binary logistic regression | Extrinsic cues significantly impact the willingness of time banking service providers to participate. Time coins return shows a significant positive effect on service transaction results (β=0.498, p<0.001), and, service hours shows a significant negative impact on service transaction results (β=-0.118, p<0.001). Intrinsic cues significantly affected willingness of time bank service providers to participate. Empathy-altruism cues have significant negative effects on service completion (β=-0.203, p<0.05), which, suggests that too much negative information can instead reduce service providers’ willingness to participate. Social cues (β=-0.5, p<0.001) and value cues (β=0.189, p <0.05) |
|  | | | |  |  |  | |  |  |
| Table 1, continued. | | | |  |  |  | |  |  |
| **No.** | **Author/Year** | **Aim** | | **Location** | **Sampling Method/Sample** | **Data Collection** | | **Design/ Measurement** | **Findings** |
| 15. | Maharsi et al. (2023) | To explain the motives, consolidation process, resource mobilization, campaign processes, social dynamics and impacts of time banks across United Kingdom | | United Kingdom | Purposive  N = 2 | Semi-structured interview and document review | | Qualitative  Critical analysis and generalization | The social challenge that Time Bank experienced was the ridicule and co-optation of the movement. Internal barriers experienced by Time Bank as an alternative social movement are members' disagreements over the redefinition of work & exchange rates, pragmatic use of applications, and relatively uniform membership composition. External barriers experienced are funding, access to public space, government policies that have a social impact, and changing services to digital. |
| 16. | Ozanne & Ozanne (2020) | To explore how time banking provides important social support helping them to cope and transition into new social roles. | | New Zealand | Snowball  N = 20 | Semi-structured interviews and observations | | Qualitative  Framework analysis | sharing skills and services in the TB provides consumers with emotional support, cognitive support to move through liminality. Some informants initially struggle to use the TB, others would like to use it more, and some members are unable to find the services they need in the TB marketplace. Some participants in the TB have difficulty moving from the motive of rational efficiency that drives marketplace exchanges. |
| 17. | Matthew (2020) | To explore the use of classroom-based experiential time banking as a community practice teaching strategy | | United States | Purposive  N = 40 | Collective and individual document review | | Qualitative  Case study  Researcher developed questionnaire | Two major themes emerged for challenges of participating timebank:   1. Lack of time 2. Lack of diverse skills/recognition of skills   Two major themes emerged for benefits of participating timebank:   1. Building senses of community 2. Capacity building/empowerment |
|  |  |  | |  |  |  | |  |  |
|  |  |  | |  |  |  | |  |  |
|  |  |  | |  |  |  | |  |  |
| Table 1, continued. | | | |  |  |  | |  |  |
| **No.** | **Author/Year** | **Aim** | | **Location** | **Sampling Method/Sample** | **Data Collection** | | **Design/ Measurement** | **Findings** |
| 18. | Naughton-Doe et al. (2020) | To explore the potential contribution of time banking and its challenges | | England | Purposive  N = 97 | Semi-structured interviews, subjective surveys and background materials | | Qualitative  Thematic analysis, framework approach | Themes:   1. Managing risk/safeguarding 2. Time banking mechanism was complex and resource intensive |
| 19. | Socci et al. (2020) | To analyse concrete experience of elderly acting as key player | | Bulgaria Denmark England France Spain | Purposive  Bulgaria (n = 5)  Denmark (n = 3)  England (n = 3)  France (n = 3)  Spain (n = 1) | Document review | | Longitudinal diary study  Thematic analysis | Themes:   1. Social entrepreneur elements 2. Social innovation elements 3. Capacity building elements |
| 20. | Schmidt (2019) | To explore the source of motivation to use sharing economy platforms | | Russia | Convenience  N = 25 | Semi-structured interview and observation | | Qualitative  Thematic analysis | Result suggest that participants on the platforms are driven by the potential of minimising transaction costs and intrinsic motivation such as getting experiences which have no market alternatives, upcycling and disposal of belongings, self-promotion and realisation. |
| 21. | Eskelinen (2018) | To examines the challenges to self-organisation and upscaling of a timebank | | Finland and United Kingdom | Convenience  Finland (n = 4-10)  United Kingdom (n = 10) | Structured interviews, observation and background materials | | Qualitative thematic analysis | Themes:   1. Government responses: market and labour 2. Governing the volunteer labour 3. Pressures from commercial agents |
|  |  |  | |  |  |  | |  |  |
|  |  |  | |  |  |  | |  |  |
|  |  |  | |  |  |  | |  |  |
| Table 1, continued. | | | |  |  |  | |  |  |
| **No.** | **Author/Year** | **Aim** | | **Location** | **Sampling Method/Sample** | **Data Collection** | | **Design/ Measurement** | **Findings** |
| 22. | Dury (2018) | To explore motivation to start, continue and quit volunteering in a time bank. | | Belgium | Convenience  N = 13 | Focus group interview | | Prospective longitudinal  Thematic analysis | The complexity of retaining volunteers stems from the fact that although initial volunteering motives are clearly defined, other contextual factors such as relationship among members and organizational structures influence the initial motives and can result in turnover. Volunteers indicated that organizational support such as co-production, meetings, incentives and attentive officers was key to staying motivated. |
| 23. | Papoikonomou & Valor (2016) | To explore commitment in peer-to-peer exchange in the case of timebanks | | Spain | Convenience  1^st^ year (n = 27}  2^nd^ year (n = 13) | Semi-structured interview | | Qualitative longitudinal  Grounded theory | Commitment is a complex and multidimensional construct that may vary drastically depending on the distinct foci of commitment, the dual roles that users perform (as providers or as recipients), the type of exchange system and the notion of reciprocity underlying the exchange system. |
| 24. | Belloti et al. (2015) | To explore challenges that hinder time banking participation. | | United States | Convenience  N = 50 | Semi-structured interview | | Qualitative  Thematic analysis | Themes and sub-themes:   1. Credit 2. Motivation 3. Relationships 4. Community |
| 25. | Molnar (2011) | To explores the organization’s: 1) challenges, 2)achievements  3) if these can be attributed to use of time banking | | Sweden | Purposive  N = 6 | Semi-structured interview  Observation | | qualitative and quantitative content analysis | Challenges identified:   1. Ineffectiveness of the Time Credit System 2. High Time Commitment 3. Difficulty in Attracting Participants 4. Lack of Socialization Among Participants |
|  |  |  | |  |  |  | |  |  |
|  |  |  | |  |  |  | |  |  |
| Table 1, continued. | | |  |  |  |  | |  |  |
| **No.** | **Author/Year** | **Aim** | | **Location** | **Sampling Method/Sample** | **Data Collection** | | **Design/**  **Measurement** | **Findings** |
| 26. | Zhao & Xu (2023) | To examine the willingness of the rural elderly to participate in time bank mutual care model. | | China | Convenience  N = 332 | Online questionnaire | | Cross-sectional  Researcher developed questionnaire | The willingness of older persons to participate in the time-banking model of mutual support for the elderly is affected by the degree of cognition, behavioural attitudes, perceived usefulness, intuitive behavioural control and subjective norms. |
| 27. | Wu et al. (2021) | To investigate the participation willingness of online timebank elderly care, especially to discover different influencing factors on the participation willingness between the youth group and the elderly group. | | China | Convenience  N = 353 | Online and self-administered questionnaire, in-depth interview | | Mixed-method  Researcher developed questionnaire  narrative analysis | 1. The health status of elderly people and the number of elder families of young people have significant positive impacts on their willingness to participate in online timebank. 2. The experience of participating in voluntary activities has a significant positive effect and it has a far greater impact in the young group than that in the elderly group. 3. The more the free time, the higher the participation willingness in the young group, but it is the opposite in the elderly group. 4. The years of education and party member have significant promoting effects on the participation willingness in both groups. |
| 28. | Valor & Papoikonomou (2017) | To examines the structure management of TBs in Spain to develops a taxonomy and identifies the goals and the reasons why users join them. | | Spain | Spain  Qualitative (N = 28)  Quantitative (N = 270) | Online questionnaire, semi-structured interviews, observations | | Mixed-method  Researcher developed questionnaire  narrative analysis | It concludes that TBs are a social-political project rather than a utilitarian-economic project, identifying differences between Spanish TBs and those in other countries |
| Table 1, continued. | | | |  |  |  | |  |  |
| **No.** | **Author/Year** | **Aim** | | **Location** | **Sampling Method/Sample** | **Data Collection** | | **Design/**  **Measurement** | **Findings** |
| 29. | Burgess (2017) | To evaluate the outcomes and challenges of time bank | | England | Cambridgeshire  (n = 47)  Wisbech (n = 22)  Stakeholders (n= 7) | Online questionnaire, semi-structured interviews, observations | | Mixed method ethnography  EQ-5D scale  Thematic analysis | Themes emerged (outcome):   1. Improvements in physical activity 2. Improvements in mental health 3. Reduced loneliness and social exclusion 4. Improved self-confidence 5. Making a positive contribution 6. Skills development 7. Work experience and pathways to paid employment 8. Strengthened family and wider relationships 9. Access to activities   Themes emerged (challenges):   1. Hoarding time credits 2. Issues around time value 3. Lack of spend activities (geographical) 4. Time credits versus money |
| 30. | Shih et al. (2015) | To understand user motivations and experiences with service exchange using time-based currency | | United States | N = 446 | Online questionnaire, semi-structured interviews, observations | | Mixed-method  Researcher developed questionnaire  narrative analysis | Findings suggest that the ideal of ‘equal time, equal value’ that is at the foundation of time banking is a source of tension between members with instrumental versus idealistic and altruistic motivations. |
