# Supplementary material for: Willingness, motivators and barriers to time bank participation: A scoping review
Source: PLoS One. 2026 Apr 1;21(4):e0322760. doi: 10.1371/journal.pone.0322760 (PMC13042674; doi:10.1371/journal.pone.0322760)
Supplement: Supplementary file 3 [file pone.0322760.s002.docx]

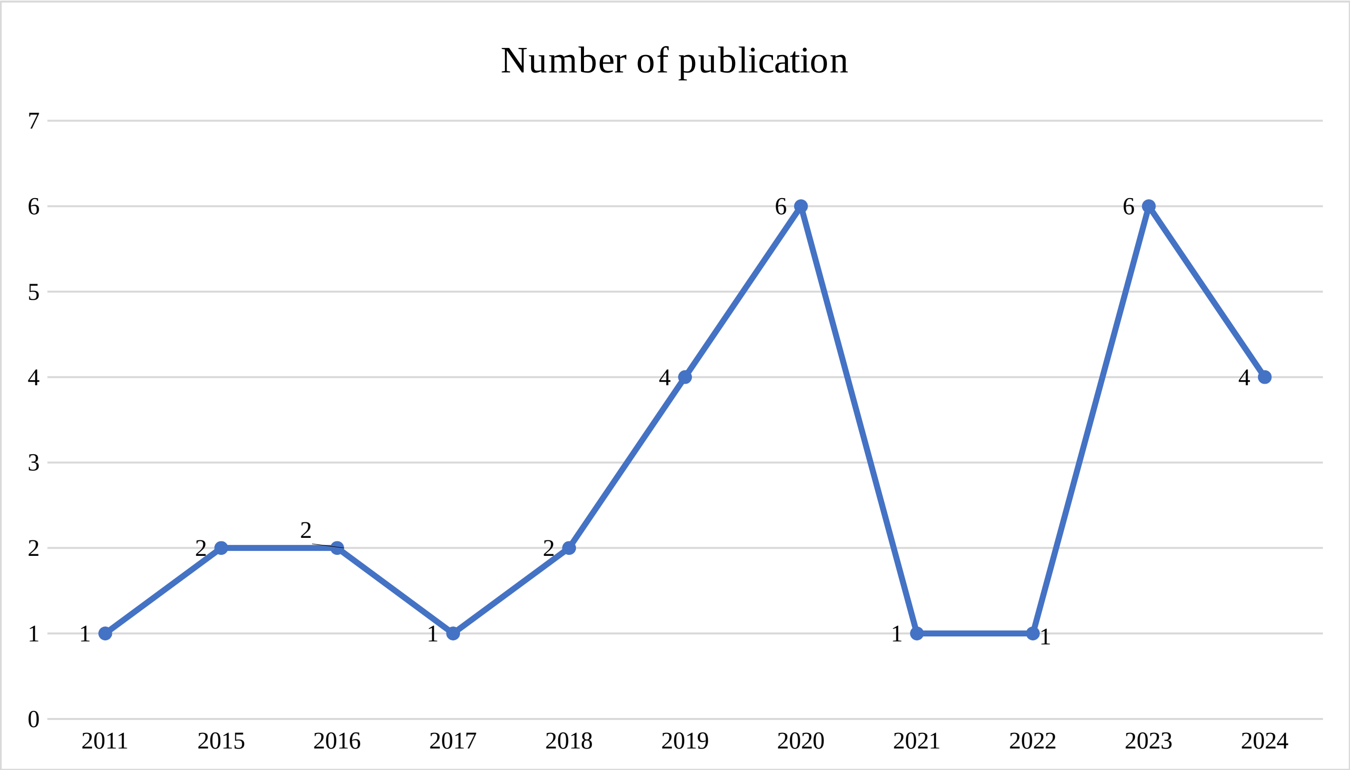

Number of publication
7
6
5
4
3
2
1
0
2011
2015
2016
2017
2018
2019
2020
2021
2022
2023
2024
1
2
2
1
2
4
6
1
1
6
4
