# Supplementary material for: Willingness, motivators and barriers to time bank participation: A scoping review
Source: PLoS One. 2026 Apr 1;21(4):e0322760. doi: 10.1371/journal.pone.0322760 (PMC13042674; doi:10.1371/journal.pone.0322760)
Supplement: Attachment — Submitted filename: Response to reviewers.pdf [file pone.0322760.s004.pdf]

PONE-D-25-16688: Willingness, Motivators and Barriers to Time Bank Participation: A Scoping Review

Dear Editor,

Thank you very much for your professionalism and consideration on our manuscript. The reviewers' constructive comments and suggestions have greatly help us improved the quality of the manuscript. We have done major revision based on the comments. We answer all of the questions or comments in details in the following summary table.

**Replies to Reviewer 1**			
**Bil.**	**Topic / subtopic**	**Reviewer's comments**	**Action taken**
1.	Abstract<br>Page 2	The abstract should exclude the headings such as objective, introduction, design etc.	We appreciate this valuable suggestion. In accordance with the guidance, we have revised the abstract to remove subheadings (e.g., Objective, Introduction, Design) and reformatted it into a single continuous narrative. The revised abstract now presents the study in a concise, coherent flow that aligns with the journal's requirements.
2.	Method<br>Section 3.4<br>Line 264-275	How the study using scoping review method addresses subjectivity bias of the authors is not discussed.	We agree that addressing potential subjectivity bias is crucial in scoping reviews. To strengthen the manuscript, we have now included a section in the Methods describing the strategies undertaken to minimize such bias. Specifically, two reviewers independently screened and charted the data, with discrepancies resolved through discussion and consensus, and intercoder agreement was sought with the supervisors. We also followed the PRISMA-ScR guidelines to ensure transparency and reproducibility. These steps were added to highlight how subjectivity bias was managed, thereby enhancing the rigor and credibility of the review.
3.	Introduction (Citations)<br>Section 2<br>Line 52-89	I believe scoping review is booted with many citations and references, however, I cannot see that in the Introduction section and other parts.	We agree that a scoping review must be firmly grounded in the existing literature. In the revised manuscript, we have substantially strengthened the Introduction and other relevant sections by incorporating a wider range of citations to situate our study within the broader body of research. Key references have been added to provide theoretical context, justify the relevance of time banking in the ageing population, and reflect current debates in social policy, gerontology, and community development. These additions ensure that the manuscript is more robustly supported by the literature and consistent with the standards expected of a scoping review.
4.	Introduction<br>Section 2<br>Line 52 - 89	The study lacks the rationale, objectives and research gap for conducting the scoping review in the introduction section.	We agree that the original Introduction section did not sufficiently emphasize the rationale, research gap, and objectives of the study. To address this, we have revised the section to provide a clearer justification for conducting the scoping review by highlighting the demographic shift toward an ageing population and the need for innovative, community-based models such as time banking to promote active ageing and social inclusion. We have also articulated the research gap, noting that despite global adoption of time banking, little evidence has been synthesized in the Malaysian or wider Asian context, particularly regarding older adults in urban communities. Furthermore, we have explicitly stated the objectives of the review, which are to map existing evidence, identify motivators and barriers to participation, and discuss implications for policy and practice. These revisions strengthen the Introduction and align it with best practices for scoping reviews.

**Replies to Reviewer 1**			
**Bil.**	**Topic / subtopic**	**Reviewer's comments**	**Action taken**
5.	Ethical Approval	Line 107, statement about ethical approval should be removed. It is not something part of the manuscript purpose itself.	We agree that the statement regarding ethical approval, as noted on line 107, is not essential to the purpose of this scoping review. Accordingly, we have removed this statement from the revised manuscript to ensure clarity and relevance.
6.	Methods Section 3.2 Line 202-216	Developing questions in the methods section requires preliminary literature, which is lacking here to support the questions.	We acknowledge that the initial draft did not sufficiently reference the preliminary literature that informed the development of the research questions. In the revised manuscript, we have strengthened the methods section by explicitly linking the formulation of the research questions to key sources from existing literature on time banking, ageing, and community-based interventions. These citations provide theoretical and empirical justification for the chosen focus areas, ensuring that the questions are clearly grounded in prior scholarship. This revision not only addresses the reviewer's concern but also enhances the methodological rigor and transparency of the study.
7.	Methods Section 3.3 Line 217 - 225	Why it leads to choose time frame January 2010 to December 2024, is not discussed	We acknowledge that the rationale for selecting the time frame of January 2010 to December 2024 was not clearly explained in the initial draft. In the revised manuscript, we have added clarification that this period was chosen because scholarly interest and formal documentation of time banking initiatives have increased substantially since 2010, coinciding with the expansion of community-based ageing strategies and the wider adoption of digital platforms supporting time banking. Extending the review until December 2024 ensures that the most recent and relevant studies are captured, thereby providing a comprehensive and up-to-date synthesis. This justification has now been explicitly included in the Methods section to enhance transparency.
8.	Methods Section 3.3 242-238	Several databases were employed together, such as; electronic databases, which were MEDLINE, Embase, CINAHL, Cochrane Library, Google Scholar and Science Direct. relevant grey literature databases were also searched such as Grey Literature Report, OpenGrey, Web of Science Conference Proceedings, government documents, academic theses/dissertations) to locate and analyze relevant studies, reports, and conference abstracts for this review. The questions are several of these such as google Scholar suffers quality check. How the authors have addressed this shortcoming?	We acknowledge that Google Scholar and some grey literature sources may vary in quality due to the absence of rigorous indexing or peer-review checks. Although Google Scholar and grey literature sources were included to capture evidence beyond peer-reviewed databases, but all records were screened using the same inclusion and exclusion criteria. No formal critical appraisal was undertaken, consistent with scoping review methodology; however, studies lacking methodological clarity or relevance were excluded. This approach ensured comprehensive coverage while reducing the risk of low-quality or non-credible sources.
9.	Methods (Supplementary document)	The authors have not focused on what actually PRISMA framework wants you to do. All page's numbers mentioned in the PRISMA checklist seems irrelevant and lack of required information.	We acknowledge that in the initial submission, some of the page numbers listed in the PRISMA checklist were not accurately aligned with the manuscript content, and certain required details were insufficiently reported. In the revised version, we have carefully re-checked the manuscript against the PRISMA-ScR framework and ensured that all checklist items are addressed with appropriate detail. Page numbers in the checklist have been corrected

**Replies to Reviewer 1**			
**Bil.**	**Topic / subtopic**	**Reviewer's comments**	**Action taken**
			to accurately reflect where each criterion is discussed in the manuscript. Furthermore, we have expanded sections where information was lacking (e.g., rationale, objectives, search strategy, and synthesis methods) to ensure full compliance with PRISMA-ScR guidelines. These revisions enhance transparency, reproducibility, and adherence to best practices for scoping reviews.
10.	Methods Section 3.3 Line 252-267	I have checked the key strings and there seems errors in it. Moreover, the use of key strings is not appropriate and this is one of the reasons the results are not clear and there may be critical articles missing. Same goes to other databases. I cannot see the use of Boolean operators and wild cards such as asterisk (*)	We acknowledge that the initial draft did not fully reflect the appropriate use of Boolean operators, truncations, and wildcards in the reported key strings, which may have contributed to ambiguities and the risk of omitting relevant articles. We acknowledge that relying solely on terms such as 'timebank', 'time bank', 'timebanking', or 'time banking' would not capture the full range of relevant literature, as some studies describe the same concept using alternative terms. To ensure comprehensive retrieval, our search strategy therefore included related keywords such as 'peer-to-peer exchange', 'time exchange', 'service exchange', and 'community exchange'. These terms were combined using Boolean operators and truncation symbols (e.g.,), and adapted to the syntax of each database. This approach minimized the risk of overlooking articles that discuss the time bank concept but employ different terminology.

**Replies to Reviewer 2**			
**Bil.**	**Topic / subtopic**	**Reviewer's comments**	**Action taken**
1.	Discussion, Strength and Limitations Section 8 Line 817-838	This article has a clear research objective and is well-organized. It systematically examines the scope and nature of evidence regarding willingness, motivations, and barriers to community participation in time bank systems, with a particular focus on older adults. The study identifies and discusses key factors that facilitate or hinder participation in time bank systems, effectively addressing gaps in the existing literature. This article demonstrates both innovation and practical relevance. However, the limited number of case studies across a wide geographic area restricts the scope of generalization and limits the ability to derive unique insights specific to certain regions.	We thank the reviewer for the encouraging feedback on the relevance of our manuscript. We appreciate the recognition that the study systematically addresses willingness, motivations, and barriers to participation in time banking, particularly among older adults, and that it contributes both innovative and practical insights to the field. We also acknowledge the limitation raised regarding the relatively small number of case studies across diverse geographic contexts. We have now emphasized this limitation more explicitly in the Discussion section and highlighted the need for future research to conduct region-specific analyses and in-depth case studies. By doing so, we aim to provide greater nuance and contextual understanding, while situating our findings within the broader global discourse on time banking.
